# Organisation of diagnosis and treatment of idiopathic pulmonary fibrosis and other interstitial lung diseases in the Nordic countries

**DOI:** 10.3402/ecrj.v2.28348

**Published:** 2015-07-01

**Authors:** Elisabeth Bendstrup, Charlotte Hyldgaard, Alan Altraja, Tone Sjåheim, Marjukka Myllärniemi, Gunnar Gudmundsson, Magnus Sköld, Ole Hilberg

**Affiliations:** 1 Department of Respiratory Diseases and Allergy, Aarhus University Hospital, Aarhus, Denmark; 2 Department of Respiratory Diseases, University of Tartu, Tallinn, Estonia; 3 Department of Respiratory Diseases, Oslo University Hospital, Oslo, Norway; 4 Department of Respiratory Diseases and Heart and Lung Center, University of Helsinki and Helsingin University Central Hospital, Helsinki, Finland; 5 Department of Respiratory Medicine and Sleep, Landspitali, The National University Hospital of Iceland, Reykjavik, Iceland; 6 Faculty of Medicine, School of Health Sciences, University of Iceland, Reykjavik, Iceland; 7 Department of Medicine Solna, Karolinska Institutet, Lung Allergy Clinic, Karolinska University Hospital, Stockholm, Sweden

**Keywords:** interstitial lung disease, idiopathic pulmonary fibrosis, organisation, diagnosis, treatment, guidelines

## Abstract

**Introduction:**

Differences in the organisation of idiopathic pulmonary fibrosis (IPF) and interstitial lung diseases (ILDs) in the Nordic countries are not well described. Diagnostic setups, treatment modalities and follow-up plans may vary due to national, cultural and epidemiological features. The aim of the present study was to describe the different organisation of diagnostics and treatment of IPF and ILD in the Nordic countries.

**Methods:**

All university and regional hospitals with respiratory physicians were invited to respond to a questionnaire collecting data on the number of physicians, nurses, patients with ILD/IPF, the presence of and adherence to disease-specific national and international guidelines, diagnosis and treatment including ILD-specific palliation and rehabilitation programmes.

**Results:**

Twenty-four university and 22 regional hospitals returned the questionnaire. ILD and IPF incidence varied between 1.4 and 20/100,000 and 0.4 and 10/100,000, respectively. Denmark and Estonia have official national plans for the organisation of ILD. The majority of patients are managed at the university hospitals. The regional hospitals each manage 46 (5–200) patients with ILD and 10 (0–20) patients with IPF. There are from one to four ILD centres in each country with a median of two ILD specialists employed. Specialised ILD nurses are present in nine hospitals. None of the Nordic countries have national guidelines made by health authorities. The respiratory societies in Sweden, Norway and Denmark have developed national guidelines. All hospitals except two use the ATS/ERS/JRS/ALAT IPF guidelines from 2011. The limited number of ILD specialists, ILD-specialised radiologists and pathologists and the low volume of ILD centres were perceived as bottlenecks for implementation of guidelines. Twenty of the 24 university hospitals have multidisciplinary conferences (MDCs). Pulmonologists and radiologists take part in all MDCs while pathologists only participate at 17 hospitals. Prescription of pirfenidone is performed by all university hospitals except in Estonia. Triple therapy with steroid, azathioprine and *N*-acetylcysteine is not used. No hospitals have specific palliation programmes for patients with ILD/IPF, but 36 hospitals have the possibility of referring patients for palliative care, mostly based on existing oncology palliative care teams; seven hospitals have rehabilitation programmes for ILD.

**Conclusion:**

There are obvious differences between the organisations of ILD patients in the Nordic countries. We call for national plans that consider the challenge of cultural and geographical differences and suggest the establishment of national reference centres and satellite collaborative hospitals to enable development of common guidelines for diagnostics, therapy and palliation in this patient group.

Differences in the organisation of idiopathic pulmonary fibrosis (IPF) and interstitial lung diseases (ILDs) in the Nordic countries are not well described. Diagnostic setups, treatment modalities and follow-up may vary due to national, cultural and epidemiological features.

During an ongoing Nordic collaboration and development of a common Nordic registry for patients with IPF and other ILDs, the differences between the six Nordic countries have become obvious. Research in the area of ILDs, especially in IPF, has been increasing in the recent years, and so has the number and size of randomized controlled trials (1–3). Unfortunately, many trials have had a negative outcome, except for those studying the drugs pirfenidone (4, 5) and nintedanib (6). Pirfenidone was the first drug developed for IPF and is now used in clinical practice in most EU countries (7). Nintedanib is the second drug developed and will probably be available during 2015.

A comparison of experiences from European centres on adherence to pirfenidone has shown that more patients discontinue pirfenidone or continue treatment at a reduced dose compared to results from the capacity (5) randomized trials (personal communication). This observation is not uncommon when comparing randomized trials to real-world settings. However, the need for high-quality and highly specialised centres has become even more evident in a rare disease like IPF with a very dismal prognosis. Thus, there will be an increasing need for ILD centres with high-quality diagnostic and treatment services to diagnose, classify and treat patients with ILD including IPF.

A PubMed search using the search terms: ‘ILD’, ‘interstitial lung disease’, ‘IPF’, ‘idiopathic pulmonary fibrosis’, ‘organization’, ‘structure’, ‘service’, ‘care’ in different combinations resulted in one study from France (8). This questionnaire-based survey showed that 20% of French pulmonologists were involved in the management of IPF patients. The survey showed that 36% of cases were discussed at a multidisciplinary conference (MDC). The 2011 international guidelines for IPF (9) were known by 67% of the pulmonologists and 84% considered them appropriate for clinical practice.

In other diseases such as asthma, existing data suggest that a structured approach to care delivery has a positive impact on outcomes at reduced costs (10). A systematic approach to asthma management undertaken in Finland has decreased morbidity, mortality and the associated direct and indirect costs (11). In the UK, the Royal College of Physicians has published a document, ‘Allergy the Unmet Need’ (12), which describes the prevalence of allergic diseases, as well as current services and training needs pertaining to allergy care.

A robust organisation of IPF and ILD treatment may facilitate earlier identification of patients as well as a more confident diagnosis, and thus earlier initiation of treatment. Furthermore, patients suited for participation in clinical trials would be identified earlier. Well-organised IPF programmes including specific nurse programmes focus on the management of side effects, adherence to treatment, palliation and advance care programmes will improve ILD and IPF services and treatment and possibly also the survival of this patient group.

This study was designed to establish a platform for a similarly structured approach to IPF and ILD management in the Nordic countries. The aim of the present study was to describe the organisation of IPF and ILD in the Nordic countries.

## Methods

A national questionnaire (see Supplementary file 1) was filled in by each author of this paper from each Nordic country (Denmark, Estonia, Finland, Iceland, Norway and Sweden). The data collected included the number of specialists in respiratory medicine and the number of hospitals with respiratory services and ILD centres in each country. Data on epidemiology of IPF and ILD in each country were included. In addition, questions on the existence of organisational plans from national health authorities, the existence of national, regional or local guidelines and if present, how the guidelines were use are being complied and a description of possible bottlenecks for their implementation were included.

Another regional questionnaire (see Supplementary file 2) was aimed at all other hospitals with a specialised respiratory department. All Nordic university hospitals (UHs) and most regional hospitals were invited to participate. The regional questionnaire was sent out to the respiratory physician in charge of ILD patients and in case of a delayed answer followed up by reminders and in some cases a telephone call. The second questionnaire collected data on the presence of guidelines, adherence to international guidelines in the field, number of physicians and nurses and estimated numbers of ILD/IPF patients in each centre, presence of specialised ILD nurses and specific nursing guidelines, presence of ILD-specific palliation, advance care planning and rehabilitation programmes. Moreover, data on diagnostic packages, presence and organisation of MDCs, and treatment strategy on pharmaceutical intervention and participation in clinical trials were collected.

Data analysis was descriptive.

## Results

**Fig. 1:**
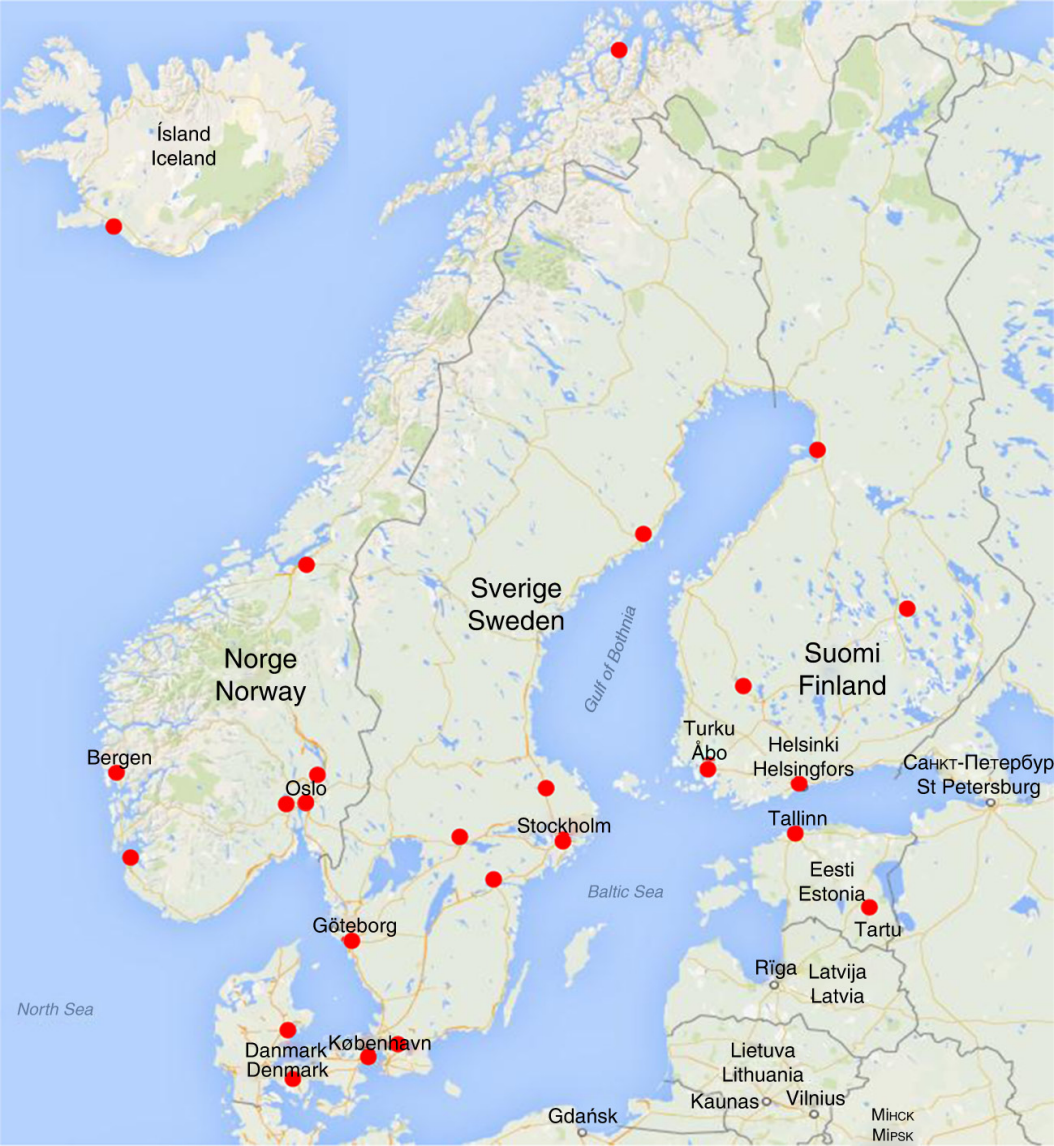
Location of the Nordic University Hospitals (red dots).

All Nordic UHs participated: Iceland 1/1 UH, Estonia 2/2 UHs, Denmark 3/3 UHs, Finland 5/5 UHs, Norway 7/7 UHs and in Sweden 6/7 UHs responded to the questionnaire (Fig. 1).

Regional hospitals also participated in the questionnaire, one from Norway, four from Sweden and 17 from Denmark. The Nordic countries each have between one and four ILD centres. The demographics are shown in Table 1.

**Table 1:** Number of inhabitants, hospitals and respiratory specialists in the Nordic countries

	Denmark	Estonia	Finland	Iceland	Norway	Sweden
Inhabitants (*n*)	5,650,000	1,315,819	5,474,094	0,326,340	5,109,059	9,694,194
University hospitals (*n*)	4	1	5	1	7	7
ILD centres (*n*)	3	2	0	1	3	4
Other hospitals^a^ (*n*)	11	9	28	0	17	35
Respiratory specialists (*n*)	130	99	200	16	180	400
Specialist/100,000 inhabitants	2.3	7.5	3.7	4.9	3.5	4.1
Inhabitants/resp. Physician	43,461	13,291	27,370	20,396	28,384	24,236

a Regional hospitals with departments of respiratory medicine.

### Incidence of ILD/IPF

The incidence of ILD and IPF in the Nordic countries is unknown. Data are based on estimates from retrospective (Denmark) or prospective studies (Finland, Iceland), on international prevalence rates (Sweden) or extrapolated data from a single hospital (Norway). ILD incidence varied between 1.4 and 20/100,000 and IPF incidence between 0.4 and 10/100,000/year.

### Overall organisation

Only two countries (Denmark and Estonia) have an official national plan for the organisation of ILDs. In Denmark, the Danish Health and Medicines Authority recommends that diagnosis and treatment of all ILD, except ‘simple’ IPF (‘simple’ is not defined), should be managed by three highly specialised centres at the UHs. At a meeting in the Danish Society of Respiratory Medicine, it was agreed that pirfenidone prescriptions should be restricted to those three centres. In Estonia, two ILD centres, one located at the UH, informally manage ILD/IPF patients. This agreement is stated in the National Development Plan for Respiratory Diseases made by the Estonian Respiratory Society for the Health Ministry. Norway, Sweden, Finland and Iceland have no official plans for diagnosis and management of ILD, but by tradition, the majority of patients are managed at UHs and larger regional hospitals. However, no complete data exist on the number of patients with ILD and IPF being managed at regional hospitals in the Nordic countries; in Denmark, 17 regional hospitals manage approximately 46 (5–200) ILD patients and 8 (0–20) IPF patients each. In Sweden, the four regional hospitals managed 39 (6–66) ILD and 13 (10–20) IPF patients according to the questionnaire responses.

### Respiratory physicians

Estonia has 7.5 respiratory physicians per 100,000 inhabitants while Denmark has only 2.3, corresponding to 13,291 and 43,461 inhabitants per respiratory specialist, respectively. Finland, Iceland, Norway and Sweden have from 3.5 to 4.9 respiratory physicians per 100,000 inhabitants, corresponding to 20,396–28,384 inhabitants per specialist. There is a median of two ILD specialists per centre in each Nordic country. Specialised ILD nurses were present in nine hospitals and six of these had specific guidelines for nurses to independently manage pirfenidone side effects.

### Guidelines

None of the six Nordic countries have national guidelines made by health authorities. The respiratory societies, one in Sweden, Norway and Denmark, have developed national guidelines. In Sweden, the guidelines for IPF and for sarcoidosis are developed by experts appointed by the Swedish Respiratory Society. In Norway, the guidelines were written after a consensus meeting with participation of respiratory specialists from the seven UHs. In Denmark, a group of experts appointed by the Danish Society of Respiratory Medicine made the guidelines. Finland has a regional guideline in one region and a few hospitals in Denmark and Finland also have local, hospital-based guidelines for the diagnosis and management of ILD and IPF. When the participants in the survey were asked to identify the bottlenecks for implementation of IPF guidelines, they mentioned the limited number of ILD specialists, ILD-specialised radiologists and pathologists and the low volume of ILD centres as problems per se and as a problem for organisation of routine MDCs. In some countries, the large number of small hospital units managing only few ILD/IPF patients was seen as bottlenecks for high-quality service.

All hospitals except two use the ATS/ERS/JRS/ALAT IPF guidelines from 2011, while only 28 of 36 hospitals use the ATS/ERS idiopathic interstitial pneumonias guidelines from 2001.

### Registries for ILD and IPF in the Nordic countries

Several ILD and IPF registries exist in the Nordic countries. Finland and Sweden have started national IPF registries in 2011 and 2014, respectively. Denmark, Norway and Iceland have registries including all ILDs except sarcoidosis, and a similar registry is planned in Estonia. All the Nordic countries collaborate on a common Nordic IPF registry based on the existing national registries.

### Diagnostic strategies and multidisciplinary discussions

The presence of a diagnostic package for newly referred patients was confirmed by 50% (12/24) of the UHs, and 50% (12/22) of regional hospitals also used diagnostic packages. A diagnostic package was defined as pre-specified investigations, such as high resolution computed tomography (HRCT), pulmonary function tests, 6-min walk test, blood sampling, echocardiography, etc., ordered routinely and not based on an individual patient assessment. Twenty of the 24 UHs had regular MDCs; at five hospitals MDCs had been introduced before 2010, while the rest had started in recent years. Eight of the regional hospitals had MDCs, in some cases as a part of a lung cancer conference. In total, 28 of 46 hospitals had MDCs. Pulmonologists (between one and six) and radiologists (between one and five) took part in all MDCs. In 17 hospitals, one pathologist participated in the MDCs and in 11 hospitals, rheumatologists participated. Nurses, clinical physiologists, thoracic surgeons, intensive care physicians, coordinators, oncologists and infectious disease specialists participated in MDCs at a limited number of hospitals.

### Treatment

Prescription of pirfenidone was performed by all UHs except in Estonia, where there is no reimbursement available yet (pirfenidone is only accessible if the patients can pay for the entire drug cost). All participating regional hospitals in Sweden and Norway prescribed pirfenidone, while none of the Danish regional hospitals did. There were differences in how drugs were reimbursed. In Denmark, the total costs are reimbursed by the health care system. In the other Nordic countries, the patients have to pay a minor part. In Norway, the physician has to apply separately for drug reimbursement for each patient. In Finland, pirfenidone can be prescribed by all physicians, but the physician has to apply for reimbursement to each patient, and patients pay an annual sum of €640 until a higher reimbursement status is granted to the drug (probably in 2015). In Iceland, only specialists in respiratory medicine can prescribe pirfenidone and in Denmark, the prescription is limited to the ILD centres. None of the 46 hospitals used triple therapy with steroid, azathioprin and *N*-acetylcysteine. Ten hospitals, including three UHs, used oral steroids as monotherapy. Before May 2014, *N*-acetylcysteine was used as monotherapy in all Danish UHs, 13/17 regional hospitals, 1/5 Finnish UHs, and 1/6 Norwegian UHs; in the UH in Iceland, in 2/6 Swedish UHs and in 1/4 regional hospitals and in no hospitals in Estonia. An interim analysis of the Panther trial (13) resulted in cessation of *N*-acetylcysteine monotherapy in most hospitals (personal communication). All Danish, three Finnish, three Norwegian and two Swedish UHs participated in pharmaceutical clinical trials.

### Palliation and rehabilitation

None of the hospitals had developed specific palliative care programmes or advance care planning specifically for IPF patients. A single centre participated in a research project on advance care planning. Almost all hospitals (36/45) had the possibility of referring patients for palliative care, mostly based on existing oncology palliative care teams. Two hospitals used oxygen nurse specialists without specific palliative education and Norway and Estonia used nursing homes. Most hospitals in Finland, Norway, Sweden, Iceland and Estonia (17/21) could refer patients for psychosocial support such as psychologists, hospital priests and social workers, while only 4/20 hospitals in Denmark had that option. Three Norwegian hospitals, two Estonian, one Swedish and the university hospital in Iceland had specific rehabilitation programmes for IPF patients, while most other hospitals referred patients to the rehabilitation programmes for chronic obstructive pulmonary disease (COPD) either at the hospital or in primary care settings. Ambulatory oxygen was prescribed by 18/20 Danish hospitals, all Finnish UHs, one Norwegian, all Estonian and Icelandic and 4/6 Swedish hospitals. Indications varied widely reflecting the lack of evidence-based guidelines (Table 2).

**Table 2:** Different indications for ambulatory oxygen in Nordic hospitals

Desaturation at 6-min walk test below 80%, 85%, 86%, 88%, or more than 4%
Improved walking distance above 30 m, 53 m or unspecified improvement
Dyspnea
Subjective improvement of dyspnea when walking with oxygen
Dizziness and headache
Reduced DLCO
Standard LTOT indication (p_a_O_2_<7.3 kPa or 60 mmHg)
Being on lung transplantation waiting list

DLCO=diffusion capacity for carbon monoxide; LTOT=long-term oxygen therapy.

## Discussion

ILDs comprise a heterogeneous group of inflammatory and fibrotic lung diseases with variable treatment responses and prognosis. IPF is the most common of the idiopathic interstitial pneumonias and is a chronic fibrotic, irreversibly progressive ILD with an estimated incidence in the Nordic countries of 0.4–10/100,000 corresponding to 1–980 new IPF patients each year in Iceland and Sweden, respectively. The majority of general practitioners will only meet 1–2 patients in their career and even respiratory physicians may only encounter few patients and thus have difficulties in achieving sufficient experience in the diagnosis and treatment of this patient group. Thus, the organisation of ILD and IPF care is important to ensure high-quality care. Other rare diseases, such as pulmonary arterial hypertension and cystic fibrosis, are organised in a few national centres in many countries, but it remains to be seen if a similar organisation can be extrapolated to the field of ILD. The optimal organisation is unknown. However, one or more national reference centre with regional competence centres may be a solution in medium- and large-sized countries, while a few reference centres in smaller countries seem rational. In France, the overall organisation is defined by a French national plan for rare diseases, in which diagnostics and management of IPF is coordinated between one national reference centre and nine regional competence centres (8). From the present Nordic questionnaire, it seems obvious that many Nordic regional hospitals diagnose and manage a small number of ILD and IPF patients. In a study from USA, it was shown that delayed access to a tertiary centre was associated with an increased risk of death independent of sex, age, pulmonary function and educational level of patients (14). Patient's delay was 2.2 years (1.0–3.8 years) in the USA (14), while Danish patients had a median duration of 13 months from patient-reported symptoms until the first visit to the referral centre (interquartile range 6–36 months) (15). Preliminary studies from the Finnish IPF registry suggest that the disease is diagnosed at a mild stage in Finland (16). Thus, early referral to an expert centre seems justified in known or suspected ILD. Moreover, early referral, diagnosis and treatment evaluation seem paramount in the light of the new treatment options with the potential of slowing disease progression.

Even though the organisation of diagnosis and treatment is heterogeneous with both ILD centres and smaller hospitals managing ILD and IPF patients, all hospitals are aware of and use the ATS/ERS/JRS/ALAT IPF guidelines from 2011 (9). Only three of the Nordic countries have specific national guidelines, but some hospitals in Denmark and Finland have national or local guidelines. On the basis of this survey, the question remains whether this is reflected in the number of patients treated with antifibrotic drugs or in long-term survival. More than half of the hospitals have a ‘diagnostic package’ with pre-specified investigations that all patients go through. It remains to be seen if this leads to an earlier and faster diagnosis, as it has been shown in patients with cancer. As IPF has the same or even a worse prognosis compared with many cancers, fast and timely diagnosis and treatment may save valuable time and resources.

A multidisciplinary approach in the diagnosis of IPF and other ILDs is recommended in the ATS/ERS statement ‘Idiopathic pulmonary fibrosis: evidence-based guidelines for diagnosis and management’ (9) and ‘Update of the International Multidisciplinary Classification of the Idiopathic Interstitial Pneumonias’ (17) as this has been shown to improve diagnostic certainty. The optimal composition of specialists in a MDC is unknown, but the statements recommend that pulmonologists, radiologists and pathologists participate. It is even recommended to refer patients to centres with MDC if a MDC is not present at their local hospital (9). Strikingly, not all UHs had routine MDCs and in many centres, MDCs were started only recently. According to the current survey, a respiratory physician and a radiologist participated in all MDCs, while pathologists only participated in less than half. A number of other specialists participated in the MDCs; it remains to be seen if the participation of other specialists will improve the diagnostic certainty.

Many hospitals refer patients with IPF to the rehabilitation programmes for COPD since specific rehabilitation programmes for patients with ILD and IPF do not exist. Studies have shown that patients benefit from participating in disease-specific rehabilitation programmes considering the disease-specific physiology into account; the disease-specific physiology in ILD is very different from that of COPD (18–20). Also, the patient education that normally forms part of any COPD rehabilitation program obviously needs to be tailored differently to meet the needs of patients with IPF. The programmes must include information on breathing patterns and exercises, specific IPF and ILD therapy, palliation, advance care planning and end-of-life decisions (18).

Palliative care programmes for IPF are sparse and the patients’ needs probably differ from those of patients with COPD and cancer (21, 22). Health care professionals often struggle with finding solutions for patients with IPF and recognize the difficulties of balancing information needs with maintaining hope. On the other hand, non-ILD professionals admit not to possess sufficient understanding of or experience with the disease (23). Therefore, a disease-specific organisation of IPF palliation is strongly needed.

In conclusion, there are many unmet needs in the care of patients with ILD and IPF. There is an obvious lack of evidence for the optimal organisation of care, although studies suggest that patients in highly specialised centres have a better survival independent of disease stage, pulmonary function and other parameters (24). Therefore, we call for national plans that take into account the challenge of cultural and geographical differences. We suggest the development of national reference centres and satellite collaborative hospitals. This strategy will enable the development of common guidelines for diagnosis and therapy, access to randomized controlled trials for all eligible patients and a common strategy for palliation including advance care planning and end-of-life decisions. Access to high-quality health care for patients with ILD is needed to improve quality of life and prognosis for these patients.

## Supplementary Material

Organisation of diagnosis and treatment of idiopathic pulmonary fibrosis and other interstitial lung diseases in the Nordic countries
