# Supplementary material for: Organisation of diagnosis and treatment of idiopathic pulmonary fibrosis and other interstitial lung diseases in the Nordic countries
Source: Eur Clin Respir J. 2015 Jul 1;2:10.3402/ecrj.v2.28348. doi: 10.3402/ecrj.v2.28348 (PMC4629764; doi:10.3402/ecrj.v2.28348)
Supplement: Organisation of diagnosis and treatment of idiopathic pulmonary fibrosis and other interstitial lung diseases in the Nordic countries [file ECRJ-2-28348-s001.zip › Appendix 1.doc]

**QUESTIONNAIRE ON** **THE ORGANIZATION OF IDIOPATHIC PULMONARY FIBROSIS AND OTHER INTERSTITIAL LUNG DISEASES IN THE NORDIC COUNTRIES**

The following questions are to be answered by the study physicians. All answers are related to January 1^st^, 2014.

**A. General questions about inhabitants, hospitals, physicians and nurses**

Data can be obtained from National registries and National Boards of Health. Remember to document your source.

1. Number of inhabitants in each country:	_____________
2. Number of specialists in Respiratory Medicine in each country:	_____________
3. Number of specialists in Respiratory Medicine per 100.000 inhabitants: _____________

(Calculation)

4. Number of University Hospitals in each country:	_____________
5. Number of other hospitals with Respiratory Medicine services:	_____________
6. Number of specialized ILD centers:	_____________
   a. Details on how the ILD center is built and organized, including referral patterns

___________________________________________________________________

___________________________________________________________________

___________________________________________________________________

___________________________________________________________________

___________________________________________________________________

___________________________________________________________________

7. Number of specialists in each Respiratory Department/University:	_____________
8. Number of doctors at each ILD managing departments involved in ILD management:	_____________
9. Number of nurses at each ILD managing departments:	_____________

**B. IPF/ILD epidemiology**

1. Estimate of ILD patients in each country and corresponding incidence (prevalence)

_____________

2. Estimate of IPF patients/country and corresponding incidence:	_____________
3. Estimate of ILD patients/University Hospital:	_____________
4. Estimate of IPF patients/University Hospital:	_____________
5. Estimate of ILD patients/specialist in the University Hospital	_____________
6. Estimate of IPF patients/specialist in the University Hospital	_____________

**C. Guidelines**

1. Has the Ministry of Health created specific plans for handling IPF and other ILD’s from an
   a. Organizational aspects, i.e. are only highly specialized centers allowed to IPF/ILD?
      1. Yes _____________	2. No _____________
   b. And if so, please describe in details how the system is organized

___________________________________________________________________

___________________________________________________________________

___________________________________________________________________

___________________________________________________________________

___________________________________________________________________

___________________________________________________________________

c. Are there national guidelines on IPF/ILD made by the health authorities?
   1. Yes _____________	2. No _____________

2. If the country is divided in regions, has the regional authorities created specific plans for handling of IPF and other ILD’s from an
   a. Organizational aspects i.e. only highly specialized centers that are allowed to deal with IPF/ILD?
      1. Yes _____________	2. No _____________
   b. And if so, please describe in details how the system is organized0

___________________________________________________________________

___________________________________________________________________

___________________________________________________________________

___________________________________________________________________

___________________________________________________________________

___________________________________________________________________

c. Are there regional guidelines on IPF/ILD supplementary to the national guidelines?
   1. Yes _____________	2. No _____________
d. Are there local (i.e. hospital based) guidelines on IPF/ILD supplementary to the national guidelines?
   1. Yes _____________	2. No _____________

3. Does the National Societies of Pulmonary Medicine have guidelines, standards or politics concerning IPF/ILD?
   1. Yes _____________	2. No _____________
4. How are national guidelines made?

___________________________________________________________________

___________________________________________________________________

___________________________________________________________________

___________________________________________________________________

___________________________________________________________________

___________________________________________________________________

5. What are the “bottle necks”, if any, for implementing the guidelines at the local level?
   a. Overall organization
   b. Pulmonologists
   c. Radiologist
   d. Pathologist

Describe the barriers:

___________________________________________________________________

___________________________________________________________________

___________________________________________________________________

___________________________________________________________________

___________________________________________________________________

___________________________________________________________________

**D. Nurses and palliation**

1. Do ILD centers/universities have ILD trained nurses?

1. Yes _____________	2. No _____________

2. And if yes, how many centers have ILD trained nurses?

_____________________________________

3. Do ILD centers have specific instructions for nurses on patient management, i.e. telephone consultations, algorithms for management of side effects of pirfenidone etc.

1. Yes _____________	2. No _____________

4. Do ILD centers have palliative care programs for ILD patients?

2. Yes _____________	2. No _____________

5. Do ILD centers have advanced care programs for ILD patients?

3. Yes _____________	2. No _____________

6. Do you have access to other palliative care programs/Advanced care programs?
   1. Yes _____________	2. No _____________
7. If yes, please describe the possibilities for palliation:

___________________________________________________________________

___________________________________________________________________

___________________________________________________________________

___________________________________________________________________

___________________________________________________________________

___________________________________________________________________

**E. Diagnosis**

1. For how many years have your ILD center existed? Since 19__ / 20__
2. Does your ILD center have a diagnostic “package” for ILD/IPF patients?

1. Yes_____________	2. No _____________

3. Did your center follow ATS/ERS guidelines from 2001?

1. Yes_____________	2. No _____________

4. Does your center follow ATS/ERS IPF guidelines from 2011?

1. Yes_____________	2. No _____________

5. Does your ILD center have multidisciplinary conferences?

1. Yes_____________	2. No _____________

6. For how many years have your ILD center had MDC? Since 19__/ 20__
7. Who participate in the conferences?
   a. Pulmonologists? Mean number of participating pulmonologists:____
   b. Radiologists? Mean number of participating radiologists:____
   c. Pathologists? Mean number of participating pathologists:____
   d. Rheumatologists? Mean number of participating rheumatologists:____
   e. Nurses? Mean number of participating nurses:____
   f. Multidisciplinary team coordinator____
   g. Physiopathologists
   h. Others? ______________________ Mean number:____

**F. IPF treatment**

1. Do you use pirfenidone?

1. Yes_____________	2. No _____________

2. When was pirfenidone accessible in your country?

________________________________________(month, year)

3. Explain the reimbursement system for pirfenidone in your country

___________________________________________________________________

___________________________________________________________________

___________________________________________________________________

___________________________________________________________________

___________________________________________________________________

___________________________________________________________________

4. Are there any specific reimbursement rules for other IPF drugs

1. Yes_____________	2. No _____________

5. If yes, please explain

___________________________________________________________________

___________________________________________________________________

___________________________________________________________________

___________________________________________________________________

___________________________________________________________________

___________________________________________________________________

6. Do you use triple therapy

1. Yes_____________	2. No _____________

7. Do you use steroid monotherapy?

1. Yes_____________	2. No _____________

8. Do you use NAC monotherapy?

1. Yes_____________	2. No _____________

9. Do you have rehabilitation programs for IPF?

1. Yes_____________	2. No _____________

10. Can you refer patients for psychosocial support?

1. Yes_____________	2. No _____________

11. Do you use ambulatory oxygen i.e. only for exercise?

1. Yes_____________	2. No _____________

12. If yes, on what indications?

___________________________________________________________________

___________________________________________________________________

___________________________________________________________________

___________________________________________________________________

___________________________________________________________________

___________________________________________________________________

13. What clinical trials are you centre involved in?

___________________________________________________________________

___________________________________________________________________

___________________________________________________________________

___________________________________________________________________

___________________________________________________________________

___________________________________________________________________

14. Are there any upper age limits for lung transplantation?

1. Yes_____________	2. No _____________

15. If yes, at what age is lung transplantation no longer performed?

________________________________________________

16. What is the mean age of IPF patients when listed on LTX list?

________________________________________________

17. Do you do single or double lung transplantation in IPF in general?

1. Single_____________	2. Double _____________
