# Supplementary material for: Organisation of diagnosis and treatment of idiopathic pulmonary fibrosis and other interstitial lung diseases in the Nordic countries
Source: Eur Clin Respir J. 2015 Jul 1;2:10.3402/ecrj.v2.28348. doi: 10.3402/ecrj.v2.28348 (PMC4629764; doi:10.3402/ecrj.v2.28348)
Supplement: Organisation of diagnosis and treatment of idiopathic pulmonary fibrosis and other interstitial lung diseases in the Nordic countries [file ECRJ-2-28348-s001.zip › Appendix 2.doc]

**QUESTIONNAIRE ON** **THE ORGANIZATION OF IDIOPATHIC PULMONARY FIBROSIS AND OTHER INTERSTITIAL LUNG DISEASES IN THE NORDIC COUNTRIES**

The following questions are to be answered by the physician in charge of ILD at the local hospital. All answers are related to January 1^st^, 2014.

A. **GUIDELINES**
   1. Are there local (i.e. hospital based) guidelines on IPF/ILD?
      1. Yes _____________	2. No _____________
2. What are the “bottle necks”, if any, for implementing the guidelines at the local level?
   1. Overall organization
   2. Pulmonologists
   3. Radiologist
   4. Pathologist
3. Describe the barriers:

____________________________________________________________________________________

____________________________________________________________________________________

____________________________________________________________________________________

**B. ORGANISATION OF YOUR CENTRE**

1. How many lung specialists are you at your ILD managing centre?	_____________

2. Number of nurses at your ILD managing department:	_____________
3. Does our ILD managing department have ILD trained nurses?
   1. Yes _____________	2. No _____________
4. Does our ILD managing department have specific instructions for nurses on patient management, i.e. telephone consultations, algorithms for management of side effects of pirfenidone etc.

1. Yes _____________	2. No _____________

5. Estimate the number of ILD patients in your ILD managing department:
6. Estimate the number of IPF patients in your ILD managing department:__________

**C. PALLIATION**

1. Does our ILD managing department have palliative care programs for ILD patients?

1. Yes _____________	2. No _____________

2. Does our ILD managing department have advanced care programs for ILD patients?

2. Yes _____________	2. No _____________

3. Does our ILD managing department have access to other palliative care programs/Advanced care programs?

1. Yes _____________	2. No _____________

4. If yes, please describe the possibilities for palliation:

_____________________________________________________________________________________

_____________________________________________________________________________________

_____________________________________________________________________________________

**C. DIAGNOSIS**

1. Does your ILD managing department have a diagnostic “package” for ILD/IPF patients?

1. Yes_____________	2. No _____________

2. Does your ILD managing department follow the ATS/ERS guidelines from 2001?

1. Yes____________	2. No _____________

3. Does your ILD managing department follow the ATS/ERS IPF guidelines from 2011?

1. Yes_____________	2. No _____________

4. Does your ILD managing department have multidisciplinary conferences (MDC)?

1. Yes_____________	2. No _____________

5. For how many years have your ILD managing department had MDC? Since 19___/ 20___
6. Who participate in the conferences?
   a. Pulmonologists? Mean number of participating pulmonologists:____
   b. Radiologists? Mean number of participating radiologists:____
   c. Pathologists? Mean number of participating pathologists:____
   d. Rheumatologists? Mean number of participating rheumatologists:____
   e. Nurses? Mean number of participating nurses:____
   f. Multidisciplinary team coordinator____
   g. Physiotherapists:____
   h. Others? Mean number:____

**D. IPF TREATMENT**

1. Do you use pirfenidone?

1. Yes_____________	2. No _____________

2. When was pirfenidone accessible at your centre?

________________________________________(month,year)

3. Do you use triple therapy

1. Yes_____________	2. No _____________

4. Do you use steroid monotherapy?

1. Yes_____________	2. No _____________

5. Do you use NAC monotherapy?

1. Yes____________	2. No _____________

6. Do you have rehabilitation programs for IPF?

1. Yes_____________	2. No _____________

7. Can you refer patients for psychosocial support?

1. Yes_____________	2. No _____________

8. Do you use ambulatory oxygen i.e. only for exercise?

1. Yes_____________	2. No _______ ______

9. If yes, on what indications?

____________________________________________________________________________________

__________________________________________________________________________________

____________________________________________________________________________________

10. What clinical trials are you centre involved in?

____________________________________________________________________________________

____________________________________________________________________________________

____________________________________________________________________________________

Thank you for your help.

Gunnar Gudmundson, Iceland	Marjukka Myllarnïemi, Finland

Giovanni Ferrari, Sweden	Alan Altraja, Estonia

Tone Sjöheim, Norway	Elisabeth Bendstrup
